# Correction: Porcine B cell receptor repertoire uncovers balanced recognition of antigenic structures on serotype Asia1 foot-and-mouth disease virus

**DOI:** 10.1371/journal.ppat.1014358

**Published:** 2026-06-22

**Authors:** The PLOS One Staff

There are errors in the author affiliations. The correct affiliations are as follows:

Shulun Huang^1,2^, Shanquan Wu^3^, Fengjuan Li^1,4^, Pinghua Li^1,4^, Pu Sun^1,4^, Yimei Cao^1,4^, Huifang Bao^1,4^, Kaiheng Dong^1,4^, Jiaxin Yang^1,4^, Hehe Zhang^1,4^, Qiongqiong Zhao^1,4^, Ying Sun^1,4^, Dong Li^1,4^, Xingwen Bai^1,4^, Yuanfang Fu^1,4^, Hong Yuan^1,4^, Xueqing Ma^1,4^, Zhixun Zhao^1,4^, Jing Zhang^1,4^, Jian Wang^1,4^, Zaixin Liu^1,4^, Yong Peng^5^, Kun Li^1,4^, Jinlian Hua^2^, Zengjun Lu^1,4^, Dongsheng Lei^1,3,6^, Qiang Zhang^1,4^

**1** State Key Laboratory of Animal Disease Control and Prevention, College of Veterinary Medicine, Lanzhou University, Lanzhou Veterinary Research Institute, Chinese Academy of Agricultural Sciences, Lanzhou, China, **2** College of Veterinary Medicine, Northwest A&F University, Shaanxi Centre of Stem Cells Engineering & Technology, Yangling, Shaanxi, China, **3** Key Laboratory of Magnetism and Magnetic Functional Materials, School of Physical Science and Technology, Electron Microscopy Centre of Lanzhou University, Lanzhou University, Lanzhou, China, **4** Gansu Province Research Center for Basic Disciplines of Pathogen Biology, Lanzhou, China, **5** School of Materials and Energy, Electron Microscopy Centre of Lanzhou University, Lanzhou University, Lanzhou, China, **6** Jiangsu Key Laboratory of Zoonosis, Yangzhou University, Yangzhou, China.

The publisher apologizes for the errors.
