# Supplementary material for: Porcine B cell receptor repertoire uncovers balanced recognition of antigenic structures on serotype Asia1 foot-and-mouth disease virus
Source: PLoS Pathog. 2026 Feb 6;22(2):e1013893. doi: 10.1371/journal.ppat.1013893 (PMC12880708; doi:10.1371/journal.ppat.1013893)
Supplement: S1 Text — Fig A. Negative-stain electron microscopy analysis of the particle integrity of purified FMDV 146S antigens before and after biotinylation. Negative-stain electron microscopy analysis of purified Asia1/JS/05 146S particles (A) and the corresponding biotinylated Asia1/JS/05 146S particles(B). Fig B. Reactivity of porcine mAbs with FMDV serotype Asia1 detected by indirect immunofluorescence assay (IFA). BHK-21 cells were infected with the Asia1/JS/05 strain and incubated with porcine mAbs (5 µg/ml), followed by incubation with FITC-conjugated rabbit anti-pig IgG (1: 200 in PBS). Fluorescence signals were observed using an FL Imaging System (Life Technologies, USA). Experiments were independently conducted in triplicate. Fig C. Reactivity of porcine mAbs with FMDV serotype Asia1 determined by indirect enzyme-linked immunosorbent assay (ELISA). Fig D. Neutralizing potency of pnAbs against FMDV Asia1/JS/05 strain and its mutants was evaluated using a microneutralization assay. The neutralizing concentration represented the minimum antibody concentration required to fully prevent CPE. The experiment was performed in triplicate. Statistical analysis was conducted by One-Way ANOVA followed by Dunnett’s multiple comparison test or unpaired T-test with a 95% confidence interval using GraphPad Prism 8.0. *, **, *** indicate significant differences from WT at P < 0.05, P < 0.01, P < 0.001, respectively. ns indicates no significant difference. Fig E. Reactivity of pnAbs PAS5 and PAS12 against denatured 146S antigen of the Asia1/JS/05 strain by Western blotting. The 146S antigen of the Asia1/JS/05 strain was denatured and reduced by heating at 100°C for 5 min in SDS-loading buffer with dithiothreitol (DTT), separated by 12% SDS-PAGE, and transferred to a methanol-activated nitrocellulose membrane. After blocking with 5% non-fat milk in TBST overnight at 4°C, membranes were sequentially incubated with porcine mAbs PAS5 or PAS12 (2 µg/ml) and HRP-conjugated anti-porcine IgG (1: 500 [file ppat.1013893.s001.docx]

**Supporting Information for**

**Porcine B cell receptor repertoire uncovers balanced recognition of antigenic structures on serotype Asia1 foot-and-mouth disease virus**

Shulun Huang^1,2,4^, Shanquan Wu^3^, Fengjuan Li^1,4^, Pinghua Li^1,4^, Pu Sun^1,4^, Yimei Cao^1,4^, Huifang Bao^1,4^, Kaiheng Dong^1,4^, Jiaxin Yang^1,4^, Hehe Zhang^1,4^, Qiongqiong Zhao^1,4^, Ying Sun^1,4^, Dong Li^1,4^, Xingwen Bai^1,4^, Yuanfang Fu^1,4^, Hong Yuan^1,4^, Xueqing Ma^1,4^, Zhixun Zhao^1,4^, Jing Zhang^1,4^, Jian Wang^1,4^, Zaixin Liu^1,4^, Yong Peng^5^, Kun Li^1,4*^, Jinlian Hua^2*^, Zengjun Lu^1,4*^, Dongsheng Lei^1,3,6*^, Qiang Zhang^1,4*^

^1^State Key Laboratory of Animal Disease Control and Prevention, College of Veterinary Medicine, Lanzhou University, Lanzhou Veterinary Research Institute, Chinese Academy of Agricultural Sciences, Lanzhou, China

^2^College of Veterinary Medicine, Northwest A&F University, Shaanxi Centre of Stem Cells Engineering & Technology, Yangling, Shaanxi, China

^3^Key Laboratory of Magnetism and Magnetic Functional Materials, School of Physical Science and Technology, Electron Microscopy Centre of Lanzhou University, Lanzhou University, Lanzhou, China

^4^Gansu Province Research Center for Basic Disciplines of Pathogen Biology, Lanzhou, China

^5^School of Materials and Energy, Electron Microscopy Centre of Lanzhou University, Lanzhou University, Lanzhou, China

^6^Jiangsu Key Laboratory of Zoonosis, Yangzhou University, Yangzhou, China

[*likun02@caas.cn](mailto:*likun02@caas.cn); [jinlianhua@nwsuaf.edu.cn](mailto:jinlianhua@nwsuaf.edu.cn); [luzengjun@caas.cn](mailto:luzengjun@caas.cn); [leids@lzu.edu.cn](mailto:leids@lzu.edu.cn); [zhangqiang@caas.cn](mailto:zhangqiang@caas.cn)

**

**

**Fig A. Negative-stain electron microscopy analysis of the particle integrity of purified FMDV 146S antigens before and after biotinylation.** Negative-stain electron microscopy analysis of purified Asia1/JS/05 146S particles (A) and the corresponding biotinylated Asia1/JS/05 146S particles(B).

**

**

**Fig B. Reactivity of porcine mAbs with FMDV serotype Asia1 detected by indirect immunofluorescence assay (IFA).** BHK-21 cells were infected with the Asia1/JS/05 strain and incubated with porcine mAbs (5 µg/ml), followed by incubation with FITC-conjugated rabbit anti-pig IgG (1: 200 in PBS). Fluorescence signals were observed using an FL Imaging System (Life Technologies, USA). Experiments were independently conducted in triplicate.


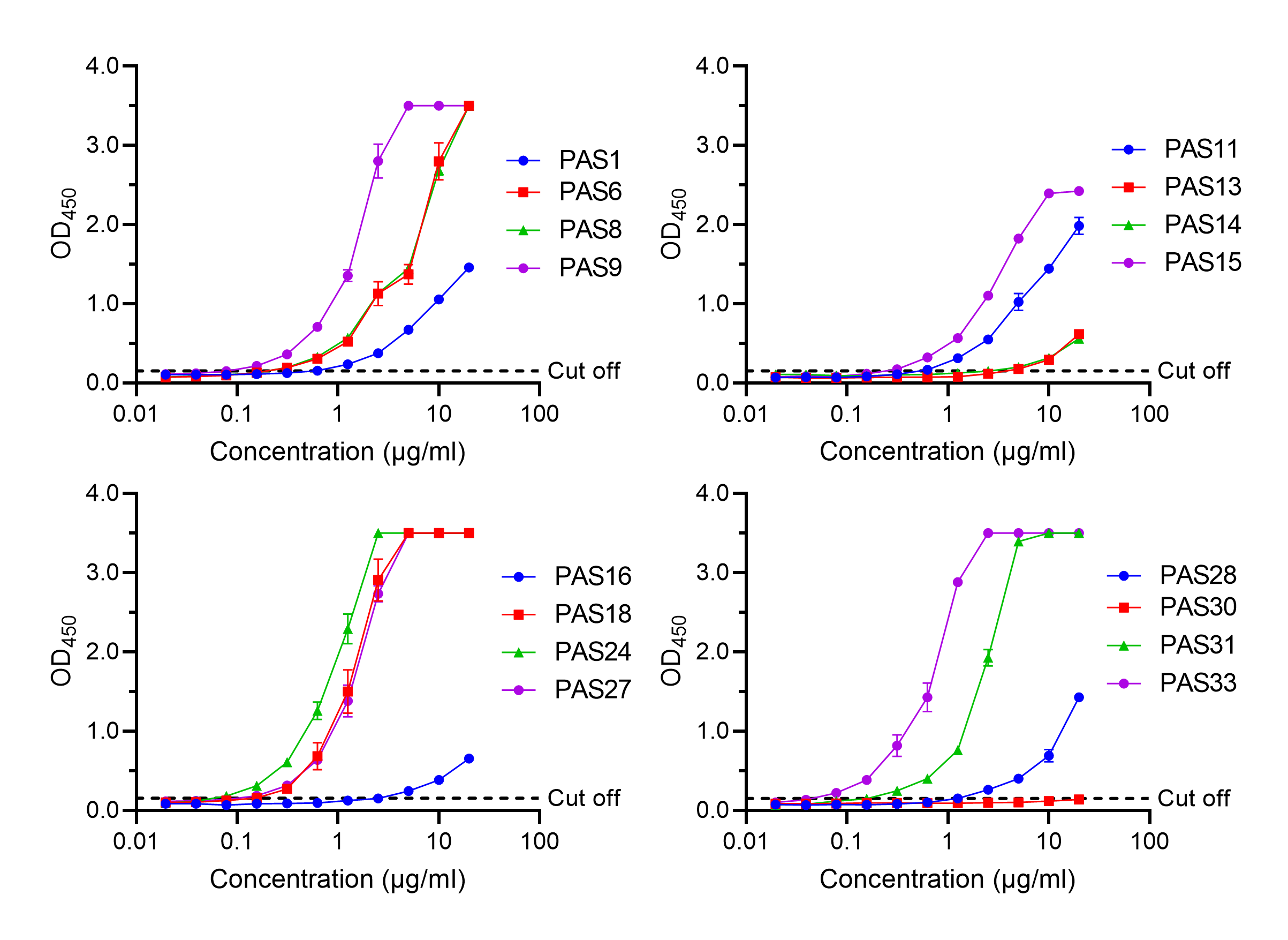


**Fig C. Reactivity of porcine mAbs with FMDV serotype Asia1 determined by indirect enzyme-linked immunosorbent assay (ELISA).**





**Fig D. Neutralizing potency of pnAbs against FMDV Asia1/JS/05 strain and its mutants was evaluated using a microneutralization assay.** The neutralizing concentration represented the minimum antibody concentration required to fully prevent CPE. The experiment was performed in triplicate. Statistical analysis was conducted by One-Way ANOVA followed by Dunnett’s multiple comparison test or unpaired T-test with a 95% confidence interval using GraphPad Prism 8.0. *, **, *** indicate significant differences from WT at P<0.05, P<0.01, P<0.001, respectively. ns indicates no significant difference.





**Fig E. Reactivity of pnAbs PAS5 and PAS12** **against denatured 146S antigen of the Asia1/JS/05 strain by Western blotting.** The 146S antigen of the Asia1/JS/05 strain was denatured and reduced by heating at 100 °C for 5 min in SDS-loading buffer with dithiothreitol (DTT), separated by 12% SDS-PAGE, and transferred to a methanol-activated nitrocellulose membrane. After blocking with 5 % non-fat milk in TBST overnight at 4 °C, membranes were sequentially incubated with porcine mAbs PAS5 or PAS12 (2 µg/ml) and HRP-conjugated anti-porcine IgG (1: 5000) for 1 h at 37 °C. Signals were visualized using an enhanced chemiluminescence solution (Thermo Fisher Scientific, USA) for 1 min and subsequently exposed to X-ray film.





**Fig F. Characterization of “SXS” and “SXR” motifs in the LCDR1 region of the Asia1/JS/05-binding BCR repertoire.** (A-B) Distributions of amino acid (AA) lengths in LCDR1 regions containing “SXS” (A) or “SXR” (B) motifs. (C) AA sequence alignment of the “SXS” and “SXR” motifs in 6-residue LCDR1 regions. Motifs were boxed in black and shaded in light blue (“SXS”) and light coral (“SXR”). (D) Usage of light chain V gene segments in 9-residue LCDR1 regions containing the “SXS” motif. (E-F) Usage of light chain V gene segments in 6-residue LCDR1 regions containing the “SXS” motif (E) and the “SXR” motif (F).





**Fig G. Plaque phenotype of wild-type (Asia1/JS/05) and rescued single-substitution mutants.** (A) Plaque formation of the WT (Asia1/JS/05) and rescued single-substitution mutants (VP2 L66A, VP2 D68A, VP2 T70A, VP2 D72G, VP2 H77A, VP2 C78A, VP2 Y80A and VP2 E195A). (B) Plaque formation of the WT (Asia1/JS/05) and rescued single-substitution mutants (VP2 L73A, VP2 S74P, VP3 E59K and VP3 K64A).





**Fig H.** **Binding modes of FMDV with integrin receptor and antibody.** The FMDV-receptor complex structure was determined by Kotecha A et al. in a previous study^1^. (A-B) Superposition of FMDV-avβ6 integrin complexes with FMDV-Asia1-PAS5 (A) or FMDV-Asia1-PAS12 (B). VP1, VP2, VP3, and VP4 of the protomer are shown in blue, green, orange, and yellow, respectively. The av and β6 chains of integrin and PAS5 or PAS12 are drawn in cartoon representation and colored in magenta, cyan, and purple, respectively. Black dashed circles show significant clashes between the antibody (PAS5 or PAS12) and the integrin receptor.





**Fig I. Sequence conservation of key antigenic sites among FMDV serotypes Asia1, A, and O.** Sequence logos illustrate amino acid conservation patterns at three major antigenic regions across representative strains of serotypes Asia1, A, and O. (A) VP2 B-C loop, (B) VP3 B-B knob, and (C) VP1 C-terminus. The height of each amino acid symbol reflects its relative frequency at that position, indicating sequence conservation or variability among serotypes. Key residues identified in this study are highlighted with red boxes.





**Fig J. Competitive ELISA analysis of sera from pigs immunized with FMDV serotypes A and O.**

Competitive ELISA was performed to evaluate the serum antibody responses targeting different antigenic sites in pigs vaccinated with FMDV serotypes A and O. (A) Archived sera from ten pigs immunized twice with the A/WH/CHA/09 vaccine were collected at 56 days post-initial immunization (dpi) and tested for competition with neutralizing monoclonal antibodies pOA-20 (Site 1), W125 (Site 2), W2 (Site 4), and W145 (Site 5). (B) Archived sera from ten pigs immunized twice with the O/HN/CHA/93 vaccine were collected at 56 dpi and tested for competition with neutralizing monoclonal antibodies pOA-20 (VP1 G-H loop), F145 (Site 2), C4 (Site 4), and B66 (VP1 C-terminus). Statistical analysis was performed using One-Way ANOVA followed by a multiple-comparison test with a 95% confidence interval in GraphPad Prism 8.0. ns indicates no significant difference. *, **, *** indicate significant differences at P<0.05, P<0.01, P<0.001, respectively.

**Table A. The interaction residues of FMDV Asia1/JS/05 with PAS5.**

| Domain | Residue | Distance(Å) | PAS5 | CDR |
| --- | --- | --- | --- | --- |
| **VP2 βB** | H65(ND1) | 3.5 | F93(CE1) | LCDR3 |
|  | L66(O) | 2.7 | N92(ND2) | LCDR3 |
|  | F67(CB) | 3.3 | N92(OD1) | LCDR3 |
|  | D68(OD2) | 3.4 | I94(N) | LCDR3 |
|  | D68(N) | 2.9 | N92(O) | LCDR3 |
| **VP2 B-C loop** | T70(OG1) | 3.7 | R96(NH2) | LCDR3 |
|  | P71(CD) | 3.2 | Y59(OH) | H-FR3 |
|  | D72(OD2) | 3.4 | R96(NH2) | LCDR3 |
|  | D72(OD2) | 3.6 | I101(N) | HCDR3 |
|  | L73(CD2) | 3.4 | W103(CZ3) | HCDR3 |
|  | H77(NE2) | 3.4 | N32(ND2) | LCDR1 |
|  | H77(ND1) | 3.0 | Y50(OH) | LCDR2 |
| **VP2 βC** | C78(O) | 3.3 | S30(OG) | LCDR1 |
|  | H79(NE2) | 3.6 | S28(O) | LCDR1 |
|  | H79(CD2) | 3.5 | N92(OD1) | LCDR3 |
|  | Y80(N) | 3.4 | S30(OG) | LCDR1 |
| **VP2 H-I loop** | V189(CG2) | 3.8 | L57(CD2) | HCDR2 |
|  | E195(O) | 2.9 | Y59(OH) | H-FR3 |

The interaction residues were computed using the CCP4^2^ hydrogen bond distance cutoff of 4.0 Å and the salt-bridge distance cutoff of 4.0 Å. The blue font refers to a hydrogen bond. The orange font refers to salt bridge.

**Table B. Interface identification and interaction analysis of PAS5 with FMDV Asia1/JS/05 by the PISA Program^3^.**

| Interface | | | | | Interaction | | | | |
| --- | --- | --- | --- | --- | --- | --- | --- | --- | --- |
| Asia1/JS/05 | **PAS5** | **BSA(Å2) ^a^** | **Percentage ^b^** | **Type ^c^** | | **PAS5^d^** | **Dist.(Å)** | **Asia1/JS/05^e^** |  |
| 2: LYS64 | L chain | 4.68 | \| | H | | L: SER30[OG] | 3.27 | 2: CYS78[O] |  |
| 2: HIS65 | L chain | 40.75 | \|\|\|\|\| | H | | L: ASN92[ND2] | 2.72 | 2: LEU66[O] |  |
| 2: LEU66 | L chain | 14.40 | \|\|\|\|\|\|\|\|\|\| | H | | L: ILE94[N] | 3.37 | 2: ASP68[OD2] |  |
| 2: PHE67 | L chain | 24.88 | \|\|\|\|\|\|\|\|\|\| | H | | L: ARG96[NH2] | 3.69 | 2: THR70[OG1] |  |
| 2: ASP68 | L chain | 53.47 | \|\|\|\|\|\|\|\|\|\| | H | | L: ARG96[NH2] | 3.44 | 2: ASP72[OD2] |  |
| 2: THR70 | L chain | 19.36 | \|\|\|\|\| | H | | L: SER28[O] | 3.59 | 2: HIS79[NE2] |  |
| 2: ASP72 | L chain | 13.87 | \|\| | H | | L: SER30[OG] | 3.37 | 2: TYR80[N] |  |
| 2: LEU73 | L chain | 13.34 | \|\|\|\|\| | H | | L: TYR50[OH] | 2.97 | 2: HIS77[ND1] |  |
| 2: SER74 | L chain | 5.83 | \| | H | | L: ASN92[O] | 2.88 | 2: ASP68[N] |  |
| 2: GLY76 | L chain | 0.56 | \| | S | | L: ARG96[NH2] | 3.44 | 2: ASP72[OD2] |  |
| 2: HIS77 | L chain | 60.81 | \|\|\|\|\|\|\|\|\| |  | |  |  |  |  |
| 2: CYS78 | L chain | 14.63 | \|\|\|\|\|\| |  | |  |  |  |  |
| 2: HIS79 | L chain | 44.02 | \|\|\|\|\|\|\|\|\|\| |  | |  |  |  |  |
| 2: TYR80 | L chain | 47.58 | \|\|\|\|\|\|\| |  | |  |  |  |  |
| 2: LEU81 | L chain | 18.07 | \|\|\|\|\|\|\| |  | |  |  |  |  |
| 2: GLU86 | L chain | 2.37 | \| |  | |  |  |  |  |
| 2: GLU131 | L chain | 26.10 | \|\|\| |  | |  |  |  |  |
| 2: GLN196 | L chain | 21.23 | \|\|\| |  | |  |  |  |  |
| 2: LYS198 | L chain | 4.77 | \| |  | |  |  |  |  |
| 2: THR70 | H chain | 19.76 | \|\|\|\|\|\| | H | | H: TYR59[OH] | 2.94 | 2: GLU195[O] |  |
| 2: PRO71 | H chain | 45.92 | \|\|\|\|\|\|\| | H | | H: ILE101[N] | 3.65 | 2: ASP72[OD2] |  |
| 2: ASP72 | H chain | 97.94 | \|\|\|\|\|\|\|\|\| |  | |  |  |  |  |
| 2: LEU73 | H chain | 13.42 | \|\|\|\|\|\| |  | |  |  |  |  |
| 2: SER74 | H chain | 24.45 | \|\|\|\| |  | |  |  |  |  |
| 2: HIS77 | H chain | 12.41 | \|\| |  | |  |  |  |  |
| 2: VAL189 | H chain | 22.32 | \|\|\|\|\|\|\| |  | |  |  |  |  |
| 2: THR191 | H chain | 5.36 | \| |  | |  |  |  |  |
| 2: GLU195 | H chain | 37.37 | \|\|\|\| |  | |  |  |  |  |
| 2: GLN196 | H chain | 11.21 | \|\| |  | |  |  |  |  |

a BSA: Buried Surface Area; b ||||: Buried area percentage, one bar per 10%. c H, Hydrogen bond; S, Salt bridge. d: Interactive atoms of amino acid side chain of PAS5. e: Interactive atoms of amino acid side chain of Asia1/JS/05.

**Table C. The interaction residues of FMDV Asia1/JS/05 with PAS12.**

| Domain | Residue | Distance(Å) | PAS12 | CDR |
| --- | --- | --- | --- | --- |
| **VP2 B-C loop** | P71(CG) | 3.7 | I30(CD1) | LCDR1 |
|  | D72(CG) | 3.5 | N54(OD1) | L-FR3 |
|  | L73(N) | 3.5 | V102(O) | HCDR3 |
|  | S74(N) | 3.4 | V102(O) | HCDR3 |
| **VP2 H-I loop** | P186(O) | 3.2 | Y104(OH) | HCDR3 |
|  | L187(O) | 3.6 | M31(CE) | LCDR1 |
|  | T188(CG2) | 4.0 | Y104(CE2) | HCDR3 |
|  | V189(N) | 3.6 | M31(SD) | LCDR1 |
| **VP3 B-B knob** | G58(O) | 3.3 | V57(CG2) | HCDR2 |
|  | E59(OE2) | 2.8 | Y33(OH) | HCDR1 |
|  | E59(OE1) | 3.5 | S52(OG) | HCDR2 |
|  | E59(N) | 3.9 | Y59(OH) | H-FR3 |
|  | V60(CG1) | 3.6 | Y106(CE2) | HCDR3 |
| **VP3 βB** | F62(CE1) | 3.7 | Y104(CE1) | HCDR3 |
|  | K64(NZ) | 3.1 | Y92(OH) | LCDR3 |

The interaction residues were computed using the CCP4^2^ hydrogen bond distance cutoff of 4.0 Å and the salt-bridge distance cutoff of 4.0 Å. The blue font refers to a hydrogen bond.

**Table D. Interface identification and interaction analysis of PAS12 with FMDV Asia1/JS/05 by the PISA Program^3^.**

| Interface | | | | | Interaction | | | | |
| --- | --- | --- | --- | --- | --- | --- | --- | --- | --- |
| Asia1/JS/05 | **PAS12** | **BSA(Å2) ^a^** | **Percentage ^b^** | **Type ^c^** | | **PAS12^d^** | **Dist.(Å)** | **Asia1/JS/05^e^** |  |
| 2: PRO71 | H chain | 11.53 | \|\| | H | | H: TYR104[OH] | 3.16 | 2: PRO186[O] |  |
| 2: ASP72 | H chain | 41.38 | \|\|\|\| | H | | H: VAL102[O] | 3.35 | 2: SER74[N] |  |
| 2: LEU73 | H chain | 0.91 | \| | H | | H: VAL102[O] | 3.50 | 2: LEU73[N] |  |
| 2: SER74 | H chain | 54.39 | \|\|\|\|\|\|\| |  | |  |  |  |  |
| 2: PHE75 | H chain | 16.84 | \|\| |  | |  |  |  |  |
| 2: GLY119 | H chain | 0.67 | \| |  | |  |  |  |  |
| 2: THR134 | H chain | 0.95 | \| |  | |  |  |  |  |
| 2: ALA185 | H chain | 4.54 | \|\| |  | |  |  |  |  |
| 2: PRO186 | H chain | 36.87 | \|\|\|\|\|\|\| |  | |  |  |  |  |
| 2: LEU187 | H chain | 2.35 | \|\|\| |  | |  |  |  |  |
| 2: THR188 | H chain | 20.26 | \|\|\|\| |  | |  |  |  |  |
| 2: PRO71 | L chain | 57.96 | \|\|\|\|\|\|\|\|\| | H | | L: MET31[SD] | 3.62 | 2: VAL189[N] |  |
| 2: ASP72 | L chain | 67.52 | \|\|\|\|\|\| |  | |  |  |  |  |
| 2: PRO186 | L chain | 4.42 | \| |  | |  |  |  |  |
| 2: LEU187 | L chain | 6.54 | \|\|\|\|\|\|\|\| |  | |  |  |  |  |
| 2: THR188 | L chain | 19.62 | \|\|\| |  | |  |  |  |  |
| 2: VAL189 | L chain | 28.97 | \|\|\|\|\|\|\|\|\| |  | |  |  |  |  |
| 2: LYS190 | L chain | 14.69 | \| |  | |  |  |  |  |
| 2: THR191 | L chain | 12.78 | \| |  | |  |  |  |  |
| 2: GLU195 | L chain | 16.26 | \|\| |  | |  |  |  |  |
| 2: GLN196 | L chain | 7.15 | \| |  | |  |  |  |  |
| 3: ARG56 | H chain | 7.28 | \| | H | | H: TYR33[OH] | 2.85 | 3: GLU59[OE2] |  |
| 3: PHE57 | H chain | 0.12 | \| | H | | H: SER52[OG] | 3.46 | 3: GLU59[OE1] |  |
| 3: GLY58 | H chain | 33.69 | \|\|\|\|\|\| | H | | H: TYR59[OH] | 3.87 | 3: GLU59[N] |  |
| 3: GLU59 | H chain | 111.38 | \|\|\|\|\|\|\| |  | |  |  |  |  |
| 3: VAL60 | H chain | 30.29 | \|\|\|\|\|\| |  | |  |  |  |  |
| 3: PHE62 | H chain | 21.78 | \|\|\|\| |  | |  |  |  |  |
| 3: LYS64 | H chain | 33.19 | \|\|\| |  | |  |  |  |  |
| 3: THR111 | H chain | 0.12 | \| |  | |  |  |  |  |
| 3: GLY195 | H chain | 15.59 | \|\|\|\| |  | |  |  |  |  |
| 3: ASP196 | H chain | 0.17 | \| |  | |  |  |  |  |
| 3: ALA197 | H chain | 10.85 | \|\|\|\|\|\| |  | |  |  |  |  |
| 3: LYS64 | L chain | 37.64 | \|\|\|\| | H | | L: TYR92[OH] | 3.12 | 3: LYS64[NZ] |  |
| 3: GLU194 | L chain | 1.73 | \| |  | |  |  |  |  |
| 1: ASP193 | H chain | 17.18 | \|\| |  | |  |  |  |  |
| 1: THR195 | H chain | 46.12 | \|\|\|\| |  | |  |  |  |  |

a BSA: Buried Surface Area; b ||||: Buried area percentage, one bar per 10%. c H, Hydrogen bond; S, Salt bridge. d: Interactive atoms of amino acid side chain of PAS12. e: Interactive atoms of amino acid side chain of Asia1/JS/05.

**Table E. Summary of FMDV-neutralizing monoclonal antibodies and their recognized antigenic sites.**

| nAb | Neutralizing spectrum of FMDV | Antigenic site |
| --- | --- | --- |
| pOA-20 | Serotype A, O, and Asia1^4^ | Site 1 (VP1 G-H loop) |
| W125 | Serotype A^5^ | Site 2 |
| W2 | Serotype A^5^ | Site 4 |
| W145 | Serotype A^5^ | Site 5 |
| F145 | Serotype O^6^ | Site 2 |
| C4 | Serotype O^6^ | Site 4 |
| B66 | Serotype O^7^ | Site 1 (VP1 C-terminus) |

**Table F. Cryo-EM data collection and refinement statistics.**

|  | FMDV-Aisa1-PAS5 | FMDV-Aisa1-PAS12 |
| --- | --- | --- |
| Data collection and processing |  |  |
| Magnification | 105,000 | 105,000 |
| Voltage (kV) | 300 | 300 |
| Electron exposure (e–/Å^2^) | 30 | 30 |
| Defocus range (μm) | -1.8 to -0.8 | -1.8 to -0.8 |
| Pixel size (Å) | 0.83 | 0.83 |
| Symmetry imposed | I1 | I1 |
| Software | cryoSPARC | cryoSPARC |
| Final particle images (no.) | 14129 | 17484 |
| Map resolution (Å) | 2.17 | 2.14 |
| FSC threshold | 0.143 | 0.143 |
| Map sharpening B factor (Å^2^) | -62.4 | -64.2 |
| Model Building and validation |  |  |
| MolProbity score | 1.67 | 1.66 |
| Clash score | 6.68 | 6.07 |
| Rotamer outliers (%) | 0.00 | 0.00 |
| R.M.S.D. of bond lengths (Å) | 0.005 | 0.003 |
| R.M.S.D. of angles (∘) | 0.639 | 0.532 |
| Favored (%) | 95.60 | 95.29 |
| Allowed (%) | 4.05 | 4.60 |
| Outliers (%) | 0.35 | 0.11 |

**SI References**

1. Kotecha A, Wang Q, Dong X, Ilca SL, Ondiviela M, Zihe R, et al. Rules of engagement between alphavbeta6 integrin and foot-and-mouth disease virus. Nat Commun. 2017;8:15408. Epub 2017/05/24. doi: 10.1038/ncomms15408. PubMed PMID: 28534487; PubMed Central PMCID: PMC5457520.

2. Winn MD, Ballard CC, Cowtan KD, Dodson EJ, Emsley P, Evans PR, et al. Overview of the CCP4 suite and current developments. Acta Crystallogr D Biol Crystallogr. 2011;67(Pt 4):235-42. Epub 2011/04/05. doi: 10.1107/S0907444910045749. PubMed PMID: 21460441; PubMed Central PMCID: PMC3069738.

3. Krissinel E, Henrick K. Inference of macromolecular assemblies from crystalline state. Journal of molecular biology. 2007;372(3):774-97. Epub 2007/08/08. doi: 10.1016/j.jmb.2007.05.022. PubMed PMID: 17681537.

4. Li F, Wu S, Lv L, Huang S, Zhang Z, Zerang Z, et al. Discovery, recognized antigenic structures, and evolution of cross-serotype broadly neutralizing antibodies from porcine B-cell repertoires against foot-and-mouth disease virus. PLoS Pathog. 2024;20(10):e1012623. Epub 2024/10/15. doi: 10.1371/journal.ppat.1012623. PubMed PMID: 39405339; PubMed Central PMCID: PMC11508087.

5. Li K, He Y, Wang L, Li P, Bao H, Huang S, et al. Conserved antigen structures and antibody-driven variations on foot-and-mouth disease virus serotype A revealed by bovine neutralizing monoclonal antibodies. PLoS Pathog. 2023;19(11):e1011811. Epub 2023/11/20. doi: 10.1371/journal.ppat.1011811. PubMed PMID: 37983290; PubMed Central PMCID: PMC10695380.

6. Li K, He Y, Wang L, Li P, Wang S, Sun P, et al. Two Cross-Protective Antigen Sites on Foot-and-Mouth Disease Virus Serotype O Structurally Revealed by Broadly Neutralizing Antibodies from Cattle. J Virol. 2021;95(21):e0088121. Epub 2021/08/19. doi: 10.1128/JVI.00881-21. PubMed PMID: 34406868; PubMed Central PMCID: PMC8513477.

7. Li K, Wang S, Cao Y, Bao H, Li P, Sun P, et al. Development of Foot-and-Mouth Disease Virus-Neutralizing Monoclonal Antibodies Derived From Plasmablasts of Infected Cattle and Their Germline Gene Usage. Front Immunol. 2019;10:2870. Epub 2019/12/24. doi: 10.3389/fimmu.2019.02870. PubMed PMID: 31867017; PubMed Central PMCID: PMC6908506.
