# Supplementary material for: Porcine B cell receptor repertoire uncovers balanced recognition of antigenic structures on serotype Asia1 foot-and-mouth disease virus
Source: PLoS Pathog. 2026 Feb 6;22(2):e1013893. doi: 10.1371/journal.ppat.1013893 (PMC12880708; doi:10.1371/journal.ppat.1013893)
Supplement: Attachment — Submitted filename: Response to Reviewers.docx [file ppat.1013893.s002.docx]

Dear editors and reviewers:

We sincerely appreciate the editor and reviewers for their valuable and constructive comments, which have greatly improved the quality of our manuscript entitled “Porcine B cell receptor repertoire uncovers immunodominant properties of antigenic structures on serotype Asia1 foot-and-mouth disease virus.”

In this revised version, we have comprehensively refined the language throughout the text to enhance clarity and readability. The Discussion section has been expanded to include a more detailed consideration of the study’s limitations.

In addition, we have performed supplementary competitive ELISA assays using sera from pigs vaccinated with FMDV serotypes A and O, providing further experimental evidence supporting the distinct immunodominance pattern of serotype Asia1.

Furthermore, Hehe Zhang, who contributed to the newly added experiments and data analysis, has been added as a co-author in the revised manuscript.

The cryo-EM density maps and structures have already been deposited in EMDB and PDB and are currently being processed for public release. We will make them publicly available before acceptance of the manuscript, in accordance with the journal’s open data policy.

Best wishes

Zengjun Lu

Response to Reviewers' Comments:

Reviewer's Responses to Questions

**Part I - Summary**

Reviewer #1: In this paper, the authors analyze the BCR repertoire of a pig immunized with and FMDV Asia1 inactivated vaccine using B-cell immune separation, expressed the mAbs in 293F cells, and determined that 17 mAbs neutralized FMDV Asia1. Isolation and sequence analysis of neutralization escape mutants revealed 3 groups: 1) 14 mAbs mapped to the VP2 B-C loop corresponding to antigenic site 2, 2) 2 mAbs mapped to the VP3 B-B knob and VP2 B-C loop corresponding to a novel antigenic site composed of site 2 and 4, and 3) 1 mAb mapped to the C-terminus of VP1. Cryo-EM analysis of mAb PAS12 form group 2 confirmed the identification of the novel epitope antigenic structure composed by VP2 and VP3 residues. The authors concluded that the VP2 B-C loop is the immunodominant epitope in FMDV Asia1. This is an interesting work that further analyzed the antibody response against FMDV Asia1 in pigs, which has not been studied in detail.

Response:

We sincerely thank Reviewer #1 for the careful reading and positive summary of our work. We are grateful for the recognition of our study’s novelty in characterizing the B-cell immune response to the FMDV Asia1 vaccine in pigs and for acknowledging the significance of identifying the VP2 B–C loop as the immunodominant epitope. We appreciate the reviewer’s encouraging comments and have carefully addressed all specific points raised below to further improve the clarity and quality of our manuscript.

Reviewer #2: The manuscript by Huang and colleagues addresses an interesting and important topic. The application of porcine B cell receptor repertoire sequencing to investigate antigenic structures of FMDV serotype Asia1 is relatively novel and has potential implications for vaccine development. However, there are several critical areas where the manuscript could be improved.

Response: We sincerely thank Reviewer #2 for recognizing the novelty and importance of our study, particularly the application of porcine B cell receptor repertoire sequencing to investigate FMDV Asia1 antigenic structures and its potential relevance for vaccine development. We appreciate the reviewer’s thoughtful feedback and have carefully addressed all specific comments below to improve the clarity, rigor, and quality of the manuscript.

Reviewer #3: Overall this is an excellent and highly valuable study that adds to our limited understanding of natural host responses to vaccine induced FMD protection. The methods are established but remain contemporary and are used to answer some very specific questions, trying to better determine the antigenic landscape of a viral capsid that has been used so effectively to eradicate the Asia1 serotype from China. Taking a comparative approach to epitope availability compared to other serotypes that remain a threat is a powerful approach and a potentially useful approach.

A clear strength of the work is the high resolution methodologies that have been used effectively to look at the molecular interactions and mechanisms of virus neutralisation. However, the overall conclusions that are drawn rely on one young pig being representative of all pigs, and in think this needs to be tempered throughout the manuscript. The clear phenotyping of antigen specific B cells and the unusual role of the light chain are very interesting and novel findings that help us to better understand protective responses in pigs, not only to FMDV.

Response:
We sincerely thank Reviewer #3 for the thorough and positive evaluation of our study. We are grateful for the recognition of the novelty and value of our work, including the high-resolution methodologies used, the detailed phenotyping of antigen-specific B cells, and the insights regarding the role of the light chain in FMDV neutralization. We also appreciate the constructive comment regarding the generalization of conclusions from a single young pig, and we have tempered our statements throughout the revised manuscript to more accurately reflect the scope and limitations of our study.

**Part II – Major Issues: Key Experiments Required for Acceptance**

Please use this section to detail the key new experiments or modifications of existing experiments that should be absolutely required to validate study conclusions.

Generally, there should be no more than 3 such required experiments or major modifications for a "Major Revision" recommendation. If more than 3 experiments are necessary to validate the study conclusions, then you are encouraged to recommend "Reject".

Reviewer #1:

Question 1

The paper requires editing of the English language as well as technical editing because it is difficult to understand the ambiguous meaning of many sentences. It is surprising that no mAbs were isolated against the VP1 140-142 immunodominant neutralizing epitope that contains the RGD receptor binding site. The authors attributed this unexpected issue to the unique structure of the FMDV Asia1 particles that differs from other FMDV serotypes and to the distinctive porcine light-kappa antibody response, explanations that are not well substantiated or convincing enough to this reviewer. Furthermore, the authors did evaluate some study design pitfalls such as the effect of the inactivation process in the antigenic structure of FMDV Asia1 or the use of only one pig in the BCR repertoire study.

Response: Yes, we have thoroughly revised the manuscript to improve English language, clarity and technical accuracy. A tracked-changes version of the revised manuscript is provided to highlight all modifications, and a clean copy has also been uploaded.

Regarding the observation that no mAbs were isolated against the VP1 140–142 immunodominant neutralizing epitope containing the RGD receptor-binding site, we believe this concern may have arisen from potential ambiguity in the last paragraph of the *Results* section (Part II). We have carefully revised this section and deleted the sentence that might have caused misunderstanding. The original text may have unintentionally implied that the absence of VP1 G-H loop-targeting antibodies was attributed to the unique structure of FMDV Asia1 particles or the distinctive porcine light-chain response, which was not our intended interpretation. Instead, these descriptions were meant to summarize general immunogenetic observations. We stated the insufficient of this study in the last paragraph of Discussion section.

To address the possible impact of the inactivation process on the antigenic structure of FMDV Asia1, we have added verification of the structural integrity of the inactivated antigen in the first part of the *Results* section, and corresponding electron microscopy images have been included in the *Supporting Information* (Figure S1); (ii) the limitation of using a single pig for the BCR repertoire analysis has been explicitly discussed in the last paragraph of Discussion section, highlighting how this may affect the generalizability of our findings.

Reviewer #2:

Question 2

1.The manuscript, including the abstract, contains frequent grammatical and syntactic issues that affect readability and clarity. Common problems include incorrect verb tenses, subject-verb agreement errors, awkward phrasing, and incomplete clauses. Even in the abstract, there are multiple issues. For example, a sentence begins with “while” but is not followed by a main clause. These problems make the manuscript difficult and at times unpleasant to read. A thorough language revision is strongly recommended before further consideration.

Response: Yes, we have thoroughly revised the entire manuscript, including the abstract, to correct grammatical and syntactic errors, improve sentence structure, and enhance overall clarity and readability. All issues related to verb tenses, subject–verb agreement, and awkward or incomplete phrasing have been addressed. A tracked-changes version of the revised manuscript has been provided to indicate all language edits, and a clean version has also been uploaded for convenience.

Question 3

2.The use of terminology, particularly around the concept of immunodominance, is inconsistent and at times confusing. Phrases such as “equal immunodominances” and “average immunodominance” are not standard and are grammatically incorrect. The term "immunodominance" is typically treated as an uncountable noun and should not be pluralized. The authors are encouraged to revise the language to reflect more conventional and precise scientific usage. Terms like “even immunodominance” or “evenly distributed immunodominance” would be more appropriate and easier to interpret.

Response: Thanks for the suggestion. We have carefully revised the terminology related to immunodominance throughout the manuscript to ensure consistency and scientific accuracy. Specifically, phrases such as “equal immunodominances” and “average immunodominance” have been replaced with more appropriate expressions, including “evenly distributed immunodominance” and “comparable immunodominance.” These revisions now conform to standard immunological terminology and improve the clarity and readability of the text.

Question 4

3. A significant limitation of the study is that the B cell repertoire analysis and monoclonal antibody discovery were based on a single animal (pig #2219). Although sera from 10 pigs were used for follow-up serological testing, the central sequencing and epitope mapping results are derived from just one individual. Since pigs are an outbred species, there is likely to be substantial variation in immune gene usage and antibody responses across individuals. This raises concerns about the generalizability of the findings. The authors should clearly acknowledge this limitation and, ideally, include additional pigs in the BCR analysis to assess the reproducibility of the observations.

Response: Yes, we acknowledge that a key limitation of our study is that the BCR repertoire analysis and monoclonal antibody characterization were conducted on a single animal (pig #2219). We have now clearly acknowledged these limitations in the last paragraph of Discussion section and emphasize that future studies incorporating multiple animals and broader repertoire coverage are warranted to fully capture the porcine humoral response to FMDV Asia1.

Question 5

4. The number and diversity of neutralizing antibodies identified also seem limited. Of the 33 monoclonal antibodies expressed, only 17 were neutralizing, and many of these shared similar VH and VL gene segments, particularly IGHV1-4 and IGKV1-11. This redundancy calls into question how representative the antibody panel is and whether it captures the full range of epitope diversity. The authors should address whether this pattern reflects a biological feature of the Asia1-specific response or is a consequence of sampling bias. They should also consider whether the current dataset is sufficient to support broad conclusions about immunodominance.

Response: Yes, we acknowledge that the number and diversity of neutralizing antibodies identified are inherently limited. Of the 33 monoclonal antibodies expressed, only 17 exhibited neutralizing activity, and several share similar VH and VL gene segments, particularly IGHV1-4 and IGKV1-11. This redundancy may reflect both biological features and technical constraints: biologically, the porcine response to FMDV Asia1 appears to be dominated by certain VH/VL gene families, as also observed in other serotypes; technically, the limited number of expressed antibodies and strict clonotype selection criteria may bias representation, potentially underestimating subdominant clonotypes and antibodies targeting epitopes such as the VP1 G–H loop. We have now explicitly discussed this limitation in the Discussion section of revised manuscript and caution that, while our dataset provides robust insights into the predominant immunodominant sites, broader conclusions about the full spectrum of epitope diversity will require additional animals and more extensive repertoire coverage.

Question 6

5. The manuscript suggests that a more evenly distributed pattern of immunodominance may contribute to the successful eradication of FMDV serotype Asia1. While this is a compelling idea, there are no comparative data from serotype O or A included under similar experimental conditions. Without such controls, it is difficult to evaluate whether the observed response is truly unique to Asia1. The inclusion of cELISA or BCR repertoire data from pigs immunized with serotype O or A would provide essential context and help strengthen the manuscript’s central claim.

Response: We thank the reviewer for this insightful comment. We agree that comparative data from pigs immunized with serotype O or A would provide valuable context. However, BCR repertoire sequencing data under equivalent experimental conditions for these serotypes are not currently available. To partially address this point, we have performed additional competitive ELISA (cELISA) assays using sera from pigs immunized with FMDV serotype O and A vaccines. These data reveal the binding profiles of antibodies targeting different antigenic sites and are now included in the revised manuscript (4^th^ paragraph of the 5^th^ *Results* section). The results (Figure S10 & Table S6 in the *Supporting information*) indicate that, for both serotypes A and O, the VP1 G-H loop (site 1) represents the primary immunodominant region, supporting a comparison with the relatively even immunodominance observed for serotype Asia1.

Question 7

6. The competitive ELISA presented in Figures 7F and 7G is central to the conclusion regarding evenly distributed immunodominance, yet the methodology for this assay is not described. Critical details are missing, including how the assay was designed, how the serum samples were diluted, and how competition was measured. As this assay supports one of the manuscript’s main conclusions, a complete and detailed description of the protocol should be included.

Response: We thank the reviewer for this valuable observation. We have now added a detailed description of the assay in the *Materials and Methods* section. Specifically, we have included information on the coating of FMDV 146S antigen, the preparation and serial dilution of pig serum samples, the pre-incubation with specific porcine monoclonal antibodies, and the calculation of competitive inhibition to quantify the relative abundance of antibodies targeting each antigenic site.

Reviewer #3: (No Response)

________________________________________

**Part III – Minor Issues: Editorial and Data Presentation Modifications**

Please use this section for editorial suggestions as well as relatively minor modifications of existing data that would enhance clarity.

Reviewer #1:

Question 8

1) Lines 117-120. This sentence needs to be edited. What the authors probably mean is that they purified IgM- B cells by negative magnetic separation.

Response: Yes. The sentence has been revised to accurately indicate that IgM⁻ B cells were enriched by depleting T cells, CD14⁺ monocytes, NK cells, and naïve IgM⁺ B cells using negative magnetic separation.

Question 9

2) Line 204. Binding affinities of all mAbs were not measured in this study so it cannot be determined if they are different. Please, change or delete this sentence.

Response: Yes. The sentence has been revised to replace “binding affinity” with “binding activity” to more accurately reflect that the differences were based on ELISA and IFA results rather than quantitative affinity measurements.

Question 10

3) Lines 233-238, Fig 3C. The ELISA procedure should be moved to the M&M section.

Response: Yes. The detailed description of the ELISA procedure originally included in the *Results* section (Lines 233–238) has now been moved to the *Materials and Methods* section for clarity and consistency.

Question 11

4) Lines 246-252. Unclear concept that should be move to the discussion section or deleted.

Response: Yes. The unclear conceptual content originally included in Lines 246–252 has been removed from the manuscript.

Question 12

5) Lines 423-425 and Figures 7F-G. it is unclear how the cELISA was performed. There is no description in M&M for this experiment. What are the positive and negative controls?

Response: For clarity, the competitive ELISA (cELISA) procedure has been fully described in the *Materials and Methods* section as shown in lines.

“cELISA was performed according to the following protocol. Briefly, 96-well plates were coated overnight at 4°C with E32, a bovine broadly reactive FMDV monoclonal antibody, at 0.5 µg/mL in carbonate-bicarbonate buffer (pH 9.6). After three washes with PBST, 146S antigen (1 µg/ml) was added and incubated for 2 h at room temperature. Plates were blocked with PBS containing 5% sucrose and 1% BSA for 1 h at 37 ℃. To determine the optimal concentration of biotinylated neutralizing antibodies (Biotin-nAbs), Biotin-nAbs were pre-diluted to 5 µg/mL and subjected to eight successive twofold serial dilutions. Plates were incubated with each dilution for 1 h at 37°C, washed, incubated with HRP-conjugated streptavidin (1: 30000) for 30 min, washed again, developed with TMB for 15 min at 37°C, stopped with 2 M H₂SO₄, and read at 450 nm. The dilution producing an OD450nm of ~2.0 was selected as the working concentration.

For competition assays, 50 µL of Biotin-nAb at twice this working concentration was mixed with 50 µL of serum (serial dilutions 1: 8-1: 1024) in antigen-coated wells and incubated for 1 h at 37 ℃. Plates were washed and detection was performed as described above. Each dilution was tested in duplicate. PBS served as the negative control and Biotin-nAb alone (OD450nm ≈ 2.0) served as the positive control. The inhibition rate was calculated as (average OD of positive control - OD of sample well) / (average OD of positive control - average OD of negative control). The highest serum dilution yielding >50% inhibition was recorded as the antibody titer.”

Briefly, PBS and Biotin-nAbs alone served as the negative and positive controls, respectively.

Question 13

6) Lines 448-458. The experiments in Figs 7F-G do not establish "equal immunodominance" of the epitopes but rather seem to indicate that the mAbs compete to some extent with the polyclonal antibodies from vaccinated pigs. It is unclear what is the meaning and relevance of these experiments as performed.

Response: Thanks for this comment. We agree that the competitive ELISA results do not directly prove equal immunodominance in a strict sense. Our intention was to provide an indirect indication of the relative distribution of antibody responses to the major antigenic sites on VP1, VP2, and VP3 in vaccinated pigs. Based on the measured competitive antibody titers, the primary and booster vaccinations elicited comparable levels of antibodies against the VP1 G-H loop, antigenic site 2 on VP2, and the novel site spanning VP2 and VP3, while the VP1 C-terminus response was consistently lower. We have revised the 5^th^ *Results* section (3^rd^ paragraph) to clarify this point, replacing “These results indicated there exists equal immunodominance of the predominant antigenic structures on VP1, VP2 and VP3 of the FMDV serotype Asia1” with “These results may suggest an approximately even pattern of immunodominance among the predominant antigenic structures on VP1, VP2, and VP3 of FMDV serotype Asia1” to reflect the indirect nature of this evidence.

Question 14

7) Discussion. The authors showed in this paper that under their experimental conditions a single immunized pig with inactivated FMDV Asia1 vaccine developed an unexpected antibody response directed not against the immunodominant VP1 140-142 site 1 epitope, which contains the RGD receptor-binding motif, but rather skewed towards site 2 and 4 epitopes. However, the authors in a previous publication (Li et al., 2024) showed that pigs immunized with FMDV serotypes O and A develop anti- VP1 140-142 antibodies that cross-neutralize serotype Asia1. Also immunized mice against FMDV Asia1 develop neutralizing antibodies against VP1 140-142 (Grazioli et al., 2013). A better discussion of these somehow contradictory results will increase the relevance of this paper. The authors also failed to discuss many other relevant issues such as how the genetic background and BCR repertoire of the only pig used in the single-cell sequencing analysis and production of mAbs may have skewed their results.

Response: We thank the reviewer for highlighting these important points. We acknowledge that antibodies targeting VP1 site 1 (140–142, RGD motif) have been observed in other reports. In our study, the competitive ELISA using serum from pig #2219—the same animal used for BCR repertoire analysis—showed that antibodies recognizing site 2 and the VP2/VP3-linked site were significantly higher than those against VP1 site 1 (3^rd^ paragraph in the 5^th^ *Results* section). This indicates that the observed immunodominance pattern may reflect individual variation in antibody responses.

We also note that only a limited subset of monoclonal antibodies was expressed and functionally characterized, which may not capture all antibodies directed against VP1 site 1. As discussed in the revised manuscript, these factors represent inherent limitations of analyzing the BCR repertoire from a single animal. We have now clarified this point in the last paragraph of *Discussion* section.

Question 15

8) How was the FMDV used in the vaccination inactivated, and what tests were performed to verify the antigenic integrity of the inactivated virus.

Response: The FMDV used for vaccination was inactivated with binary ethylenimine (BEI) for 28 h at 30 °C, as described in the Materials and Methods (“Virus production, purification and biotinylation of 146S antigen”). To confirm the antigenic integrity of the inactivated virus, transmission electron microscopy was performed, and representative micrographs have been newly included in Supporting information (Figure S1).

Reviewer #2: (No Response)

Reviewer #3: General comments that I think would improve the manuscript.

Question 16

The language throughout the document needs editing to make the text clearer and grammatically accurate.

Response: Yes. We have thoroughly revised the manuscript for language clarity and grammatical accuracy throughout. Sentences with awkward phrasing, incorrect verb tenses, and subject-verb agreement errors have been corrected to improve readability and ensure precise scientific expression.

Question 17

The text in the figures was usually too small in the reviewers PDF version.

Response: Thanks for the comment. We have adjusted the font size in the figures where the text was too small to improve readability throughout the manuscript.

Question 18

The figure legends would benefit from revision and more specific language throughout.

Response: Yes. We have revised the figure legends for all main figures (Figures 1–7) to improve clarity and specificity. The updated legends now use more explicit descriptions of experimental design, labeling, and statistical methods while maintaining consistency across figures.

Pigs have a relatively unique pathology after FMDV infection compared to ruminants. Some information in the introduction related to

Question 19

Response: Thanks for the suggestion. We have revised the Introduction section (3^rd^ paragraph) to include additional information describing the unique pathological characteristics of pigs following FMDV infection compared to ruminants.

Question 20

Comments that should be addressed prior to publication.

1. Can you be more explicit about the selection criterion applied to the antibodies to selected candidates for cryoEM? For example, the antibody that binds the C terminus of VP1 (PAS32) was not pursued for structural analysis.

Response: We thank the reviewer for this helpful comment. Antibodies were selected for cryo-EM analysis based on two main criteria: (i) potent neutralizing activity against FMDV Asia1/JS/05 and (ii) recognition of distinct antigen sites according to primary results by selecting neutralization escape mutants.
This information has now been included in the **“**Cryo-EM sample preparation and data collection” section of the Materials and Methods.

Question 21

2. There is a clear difference based on epitope dominance between the monoclonal antibodies and the serum/competitive ELISA. It should be noted that the vaccinated animals were older than the piglet used for B cell isolation and it would be useful to understand if there was no gross difference in the overall response of these animals, repertoire development etc.

The equal dominance of the antigenic sites, after boost perhaps suggests that there is dominance during the first encounter? Without avidity measurements for the monoclonal antibodies, the competitive ELISA from one animal is very hard to interpret more broadly. Taken together, I think the overall conclusion needs to be modified to reflect this is a pilot approach and may not be representative.

Response: Thanks for the comments. We agree that the age difference between the piglet used for B cell isolation and the vaccinated pigs used for serum analysis may contribute to variations in antibody repertoire composition and epitope dominance. The difference observed between monoclonal antibody mapping and serum competitive ELISA results may also reflect individual variation of the animal used for BCR library construction. To address this, we performed a similar competitive ELISA using the serum collected from the same pig on the day of B cell isolation (3^rd^ paragraph in the 5^th^ *Results* section). The results showed that antibodies targeting site 2, the cross-site between site 2 and site 4, exhibited higher titers than those against site 1, as described in the main text. The limitation of using a single animal for BCR repertoire construction has also been discussed in the last paragraph of *Discussion* section.

We further agree that the approximately equal recognition of antigenic sites observed after booster vaccination may reflect a dynamic reshaping of the antibody response, potentially following an initial dominance of specific sites during primary immunization. To address this, we have added a statement in the *Discussion* (2^nd^ paragraph) noting that potential influences of host age and immunization history on shaping epitope dominance could not be addressed in the present study, and that future investigations involving animals of different ages and immunization stages will be valuable to clarify how these factors affect the dynamics of antibody responses and immunodominance patterns.

Regarding the absence of avidity measurements for the monoclonal antibodies, we acknowledge that this limits the broader interpretation of the competitive ELISA data. Accordingly, we have added a statement in the final paragraph of the *Discussion* (last paragraph) to clarify that this study represents a pilot exploration of antigen-specific B cell responses in pigs, and that further analyses with larger sample sizes will be necessary to validate the generality of these observations.

Question 22

3. As a clear purpose of the paper is to identify epitopes that could support vaccine design across serotypes, the comparative analysis between the identified epitopes and the key serotypes that remain a threat is lacking. This may be an alignment or some in silico modelling approach, I am not proposing any more animal work. I think this would greatly improve the overall significance of the work and could better support the claims around immunogenicity differences and how this work could lead to better vaccines.

Response: We thank the reviewer for this valuable suggestion. In response, we have performed a comparative sequence analysis to assess the conservation of the identified antigenic sites across FMDV serotypes Asia1, O, and A. The results have been added to the revised manuscript (1^st^ paragraph in the 5^th^ *Results* section). As shown in the new figure (Figure S9 in Supporting information), residue 68 on VP2, as well as residues 206 and 209 on the VP1 C-terminus, were highly conserved. In contrast, residues 72, 73, and 77 in the VP2 B–C loop and residue 59 in the VP3 B–B knob exhibited significant serotype-specific variation, including neighboring amino acids. These regions highlight antigenic differences among serotypes and may represent potential targets for cross-serotype vaccine design. Further structural modeling based on these variable regions may provide complementary insights into cross-serotype antigenic relationships and will be considered in future work.

Question 23

4. The statement in the abstract and repeated in other parts of the manuscript stating that this knowledge "may facilitate the selection of a broader range of germline gene segments and enhance antibody diversity production, potentially conferring exceptional immunogenicity of Asia1 vaccine for viral eradication" is not linked to the findings in the manuscript or the discussion. It would benefit the paper if the authors were more explicit about this potential based on the antibodies that they eventually identified.

Response: Yes. We agree that the previous phrasing overstated the implications of our findings. We have revised the Abstract and corresponding sections in the manuscript to reflect conclusions directly supported by our data. Specifically, we now state that “The evenly distributed antigenic recognition across VP1, VP2, and VP3 of FMDV serotype Asia1 reflects a diversified antibody response that may contribute to effective neutralization and protection,” which describes the observed immunodominance pattern without extrapolating to broader germline usage or enhanced antibody diversity. As we do not have comparable BCR sequencing data from pigs immunized with serotype O or A, we cannot infer differences in antibody diversity induced by other serotypes. Accordingly, the statements in the manuscript have been modified to avoid unsupported generalizations.

Question 24

5. Line 192. Clustering B cells based on 100% identity of the heavy chain is very strict when you state clearly that the IgG compartment has undergone significant mutation and will no doubt be maturing. This may in part explain why the number of epitopes that you identified from the relatively middle to low frequency clusters is limited and it does not appear that you have studied any non-neutralising binding antibodies. From Table 3D it would appear that half of your antibodies are non-neutralising? Considering the above, do you think it is correct to say that this is the repertoire of antigen specific B cells as I am not sure evidence is provided, but assumed from the sorting. In addition, I therefore think the epitopes identified are not exhaustive. The data is excellent, the interpretation needs to be refined.

Response: Thanks for the valuable comments. How to define the clonotype to explain the mapping relationship between antibodies and epitopes is our next study. We acknowledge that our strict definition of clonotypes and the focus on expressing only high-frequency clonotypes constitute limitations of the study. While non-neutralizing antibodies are indeed part of the BCR repertoire, this study primarily aimed to characterize the antigenic structures recognized by neutralizing antibodies, as these are the key contributors to protective immunity. Regarding the antigen specific B cell repertoire, the library was generated by sorting B cells using FMDV serotype Asia1 as the bait antigen. Importantly, over 90% of the expressed clones demonstrated specific binding to Asia1, supporting that the repertoire is antigen-specific. We also agree that the identified epitopes are not exhaustive. This limitation arises from both the finite library size and expression capacity, as well as the stringent clonotype definition. We have further discussed these constraints in the revised *Discussion* section (last paregraph).
