# Supplementary material for: Porcine B cell receptor repertoire uncovers balanced recognition of antigenic structures on serotype Asia1 foot-and-mouth disease virus
Source: PLoS Pathog. 2026 Feb 6;22(2):e1013893. doi: 10.1371/journal.ppat.1013893 (PMC12880708; doi:10.1371/journal.ppat.1013893)
Supplement: Attachment — Submitted filename: Response to Reviewers_R2.docx [file ppat.1013893.s003.docx]

Dear editors and reviewers:

Thank you for your letter and the reviewers' comments on our manuscript titled *"Porcine B cell receptor repertoire uncovers immunodominant properties of antigenic structures on serotype Asia1 foot-and-mouth disease virus."* We greatly appreciate the constructive feedback, which has significantly contributed to the revision and improvement of our paper.

After two rounds of revision, we have revised the title to "*Porcine B cell receptor repertoire uncovers balanced recognition of antigenic structures on serotype Asia1 foot-and-mouth disease virus*." This change better aligns with the findings of the study.

In this revised manuscript, we have thoroughly refined the language throughout the text to enhance clarity and readability. Additionally, we have clarified in both the Abstract and Discussion that this study is a preliminary exploration. In the Introduction, we replaced an inappropriate reference with a more suitable one, as suggested by the reviewers. We also acknowledged the potential limitations of the cELISA format and adjusted the discussion of "immunodominance" throughout the manuscript to minimize the risk of over-interpretation.

Furthermore, all figures have been carefully reviewed and adjusted using PLOS’s NAAS tool. A summary of the main corrections made, along with our responses to the editors' and reviewers' comments, is provided below.

Finally, regarding the data availability issue raised during the **technical check**: We have contacted the EMDB/PDB repositories and confirmed that the accession numbers provided in the manuscript (PDB 9MQ0, 9MPZ; EMD-48509, EMD-48508) are valid and correct. The current inability to publicly access the data is due to a processing delay at the repository following the recent holidays, although a formal release request was submitted on January 7. To facilitate the review process and prove the validity of our submission, we have uploaded the Official Validation Reports for all reported structures (categorized as "Other" files in the submission system). We trust that these reports will be sufficient for your verification. We expect the data to be fully released to the public very soon.

Best wishes

Zengjun Lu

**Additional Editor Comments:**

Please check if the title may be improved after two rounds of the manuscript revision.

Response: Thanks. The title has been revised from "Porcine B cell receptor repertoire uncovers immunodominant properties of antigenic structures on serotype Asia1 foot-and-mouth disease virus" to "Porcine B cell receptor repertoire uncovers balanced recognition of antigenic structures on serotype Asia1 foot-and-mouth disease virus".

**Reviewers' Comments:**

Reviewer's Responses to Questions

**Part I – Summary**

Please use this section to discuss strengths/weaknesses of study, novelty/significance, general execution and scholarship.

Reviewer #2: The authors have addressed my previous comments, although not as fully as I had hoped. Overall, the revisions are acceptable. All reviewers raised concerns about the representativeness of a BCR repertoire generated from a single animal, and the authors now acknowledge this limitation in the Discussion, which is appropriate.

In their response to Question 21, the authors state that "this study represents a pilot exploration of antigen-specific B cell responses in pigs." However, the manuscript itself does not clearly present the work as a pilot study. I strongly recommend that the authors explicitly include the word "pilot" or "preliminary" in the Discussion or even in the Abstract. This would more accurately reflect the scope of the study.

Although I believe the authors' conclusions are likely correct, the reliance on a single pig for BCR sequencing means that the results could differ if a different animal were used. Emphasizing that this is a preliminary study will help ensure that readers interpret the findings with the proper context and caution.

Response: Thank you for your feedback and for acknowledging the revisions made in the manuscript. We appreciate your comment regarding the representativeness of the BCR repertoire generated from a single animal.

We have explicitly incorporated the term “preliminary exploration” in both the Abstract and the Discussion to better reflect the study’s nature.

In the Abstract, we revised the text to: “Herein, we present a preliminary exploration of Asia1-binding B-cell receptor repertoire, …”.

Additionally, we updated the final Discussion paragraph to: “As such, this study should be regarded as a preliminary exploration, and future studies involving multiple animals and broader repertoire coverage are warranted to gain a more complete understanding of porcine humoral responses to FMDV Asia1.”

Reviewer #3: The authors have addressed all the comments well, which does directly address the limitations of the study.

Response: Thank you for your positive feedback and for recognizing that we have addressed the study's limitations effectively. We appreciate your thorough review and are glad that the revisions have met your expectations.

Reviewer #4: The studies by Shun Huang et al., described in this revised manuscript have analysed the production of neutralising antibodies in pigs against a foot-and-mouth disease virus (FMDV) vaccine, serotype Asia1. The authors characterised the repertoire of antibodies produced from a single pig and identified the features of the virus that these antibodies recognise. These sites were largely shared with those identified previously in other studies on FMDV but it is useful to have this information for pigs. The authors suggest that the sites vary in their dominance between that observed with other serotypes. It is proposed that these results may assist in rational vaccine design.

Response: We are grateful for the reviewer’s summary and positive evaluation. As the reviewer correctly noted, while the binding sites may be shared, characterising the specific antibody repertoire and dominance directly in the natural host is essential. We are pleased that the reviewer finds these results valuable for assisting rational vaccine design.

**Part II – Major Issues: Key Experiments Required for Acceptance**
Please use this section to detail the key new experiments or modifications of existing experiments that should be absolutely required to validate study conclusions.

Generally, there should be no more than 3 such required experiments or major modifications for a "Major Revision" recommendation. If more than 3 experiments are necessary to validate the study conclusions, then you are encouraged to recommend "Reject".

Reviewer #2: (No Response)

Reviewer #3: No

Reviewer #4: 1) It is regrettable that the antibody repertoire is only determined from a single pig, it is not, therefore, certain that the immune responses from this animal are representative of the pig population, the study would be greatly strengthened by analysing one or more further, unrelated, animals. The authors do indicate that this is a limitation of their study, after the comments from other reviewers.

Question 1

Response: Thank you for your insightful comment. We agree that the use of a single pig may not fully represent the broader pig population, and that including additional unrelated animals would certainly enhance the robustness of the study. As you pointed out, we have acknowledged this limitation in the revised manuscript. We have also explicitly stated that this study serves as a preliminary investigation and that future studies, incorporating multiple animals, will be necessary to validate our findings and expand on the understanding of the immune response in pigs.

Question 2

2) The serological responses of 10 pigs to the Asia1 vaccine was assessed in a competition ELISA. However, it may be that the process of coating the FMDV antigen onto ELISA plates may change its surface properties and perhaps some sites may be less well represented than in the native 146S particles. This may make the assessment of site dominance problematic. The authors should at least comment on this. I think it would be better if the discussion of the relative dominance of these sites was much reduced.

Response: We greatly appreciate the reviewer’s insightful comment. The reviewer is absolutely correct that maintaining the native conformation of the 146S particle is crucial for accurately assessing epitope dominance, and that direct surface adsorption could alter the antigen properties.

To mitigate the risk of capsid dissociation or denaturation often associated with direct coating (e.g., exposure to high pH carbonate buffers), we employed a capture ELISA format. Specifically, we used the bovine monoclonal antibody E32 to immobilize the antigen, aiming to present the 146S particles in a more native, fluid-phase-like state. However, we acknowledge that this method has its limitations. As the reviewer pointed out, the immobilization process may introduce bias. We recognize that antigen immobilization can induce potential conformational changes, and we have addressed this limitation in the Discussion section (2rd paragraph): "However, we acknowledge that the cELISA format used in this study may affect epitope accessibility, due to potential conformational changes or steric hindrance introduced by the capture antibody (mAb E32). Therefore, definitive confirmation requires further validation using solution-phase assays or structural techniques."

Additionally, we have reduced the discussion of epitope dominance to avoid over-interpretation, as requested. In the Discussion (2rd paragraph), we have used more objective language: "In contrast, FMDV serotype Asia1 elicited a more balanced antibody response targeting antigenic sites on VP1, VP2, and VP3. This suggests a potentially even distribution of immunodominance across viral surface, which may facilitate the utilization of a broader range of germline gene segments for recognizing diverse epitopes." Furthermore, we have made appropriate modifications in the Abstract, Introduction, and Results sections to align with the revised interpretation.

Question 3

3) The authors cite reference 4 for the eradication of Asia1 FMDV in China but the reference is actually focussed on serotypes O and A, an alternative citation should be used. It is important to know whether the successful eradication of Asia1 in China included widespread vaccination of both pigs and cattle. This has implications for the utility of understanding the immune response repertoire to FMDV antigens in pigs.

Response: We thank the reviewer for this constructive comment. We have replaced the original reference 4 with the article titled "An overview of control strategy and diagnostic technology for foot-and-mouth disease in China". As detailed in the section “The main FMD control measures in China” of this paper, it explicitly discusses the mandatory vaccination strategy, confirming the widespread use of inactivated Asia1 vaccines in cattle, goats, and sheep.

Regarding the vaccination of pigs: It is important to note that pigs were generally not included in the comprehensive mandatory vaccination campaign against Asia1 at that time. This policy aligns with historical epidemiological data. As reported in another study, "Epidemiology of Serotype Asia 1 Foot-and-Mouth Disease Virus in China", which analyzed outbreaks from 2005 to 2009: "Cattle were infected in all the 46 outbreaks, whereas other species (swine, goats, and sheep) were affected in only 3 (6.5%) of the 46 outbreaks." Given the lower clinical incidence in swine during that period, vaccination efforts prioritized ruminants.

However, the exclusion of pigs from routine Asia1 vaccination creates a potential "immunity gap." Since pigs are susceptible amplifying hosts for FMDV, investigating their immune response is critical for future preparedness. Furthermore, as we emphasized in the Introduction, pigs possess distinct immunological features compared to ruminants:

"In addition to these genetic distinctions, pigs display unique pathological features following FMDV infection. Unlike ruminants, pigs develop more severe clinical disease, marked by high viral excretion during the acute phase and efficient viral clearance without progression to a carrier state. These species-specific differences in viral pathogenesis are accompanied by distinct B cell repertoire composition and immune response kinetics, which may influence antibody maturation pathways and epitope recognition."

Therefore, characterizing the porcine BCR repertoire fills a critical knowledge gap that cannot be addressed by studies on cattle or mice.

**Part III – Minor Issues: Editorial and Data Presentation Modifications**
Please use this section for editorial suggestions as well as relatively minor modifications of existing data that would enhance clarity.

Reviewer #2: (No Response)

Reviewer #3: No

Question 4

Reviewer #4: a) The use of English is still poor in places although the sense can usually be followed.

Response: Thank you for your comment. We have thoroughly revised the manuscript and improved the overall language to enhance readability.

Question 5

b) Line 202, the text refers to “Asia1-specific BCR”, as far as I understand it, the methodology does not really demonstrate specificity for Asia!, this was just the reagent used in the screening. Perhaps some of the BCRs would have also recognised other FMDVs since cross-serotypic antibodies have been identified previously.

Response: Thanks. We have replaced "Asia1-specific BCR" with "Asia1-binding BCR" in Line 202. Additionally, we have verified and corrected all parallel instances throughout the text to maintain strict scientific accuracy.

Question 6

c) Lines 314-315 (and maybe elsewhere). Mutations occur in nucleic acids not in amino acid sequences, the changes in protein sequence should be referred to as substitutions.

Response: Thank you for your suggestion. We have corrected the text in Lines 314-315 and conducted a thorough review of the manuscript, replacing "mutation" with "substitution" wherever it refers to changes in the amino acid sequence.
